# Computed-tomography based scoring system predicts outcome for clinical lymph node-positive patients undergoing radical cystectomy

**DOI:** 10.1590/S1677-5538.IBJU.2021.0329

**Published:** 2021-09-10

**Authors:** Lennert Eismann, Severin Rodler, Alexander Tamalunas, Gerald Schulz, Friedrich Jokisch, Yannic Volz, Paulo Pfitzinger, Boris Schlenker, Christian Stief, Olga Solyanik, Alexander Buchner, Tobias Grimm

**Affiliations:** 1 Ludwig-Maximilians-University Department of Urology Munich Germany Department of Urology, Ludwig-Maximilians-University, Munich, Germany; 2 Ludwig-Maximilians-University Department of Radiology Munich Germany Department of Radiology, Ludwig-Maximilians-University, Munich, Germany

**Keywords:** Urinary Bladder Neoplasms, Tomography, X-Ray Computed, Lymph Node Ratio

## Abstract

**Purpose:**

Contrast-enhanced CT scan is the standard staging modality for patients with bladder cancer undergoing radical cystectomy (RC). Involvement of lymph nodes (LN) determines prognosis of patients with bladder cancer. The detection of LN metastasis by CT scan is still insufficient. Therefore, we investigated various CT scan characteristics to predict lymph node ratio (LNR) and its impact on survival. Also, pre-operative CT scan characteristics might hold potential to risk stratify cN+ patients.

**Materials and Methods:**

We analyzed preoperative CT scans of patients undergoing RC in a tertiary high volume center. Retrospectively, local tumor stage and LN characteristics such as size, morphology (MLN) and number of loco-regional LN (NLN) were investigated and correlation to LNR and survival was analyzed. CT scan characteristics were used to develop a risk stratification using Kaplan-Maier and multivariate analysis.

**Results:**

764 cN0 and 166 cN+ patients with complete follow-up and imaging data were included in the study. Accuracy to detect LN metastasis and locally advanced tumor stage in CT scan was 72% and 62%. LN larger than 15mm in diameter were significantly associated with higher LNR (p=0.002). Increased NLN correlated with decreased CSS and OS (p=0.001: p=0.002). Furthermore, CT scan based scoring system precisely differentiates low-risk and high-risk profiles to predict oncological outcome (p <0.001).

**Conclusion:**

In our study, solely LN size >15mm significantly correlated with higher LNR. Identification of increased loco-regional LN was associated with worse survival. For the first time, precise risk stratification based on computed-tomography findings was developed to predict oncological outcome for clinical lymph node-positive patients undergoing RC.

## INTRODUCTION

Bladder cancer (UCB) represents 3% of all malignant diagnosis worldwide with a predominance in men (1). The 5-year survival rate for all disease stages is documented with 77.1% but only 4.6% for metastatic disease (1). Despite new technologies in diagnostics, surgeries and medical treatment no decrease in mortality could be noted in the last decade (1). At the time of diagnosis up to 30% are muscle-invasive carcinomas (MIBC) and are recommended to be treated by radical cystectomy (RC) with lymphadenectomy (LAE) (1, 2). Depending on tumor stage chemotherapy can be performed in a neoadjuvant or adjuvant setting to reduce risk of recurrence (2). In the case of disseminated disease chemotherapy or alternatively immunotherapy are standard of care (2).

Prognosis after RC mainly depends on tumor stage and (LN) status (2). LN metastasis are noted to be found in 15-35% of all RC specimens (3). 5-year recurrence-free survival (RFS) for all patients undergoing RC for UCB is reported with 58% in contrast to LN positive patients with 34-43% (2). Beside conventional TNM classification for LN staging the ratio of positive lymph nodes to total number of removed LN (LNR) has been reported to precisely risk stratify patients in regard of oncological outcome (4, 5).

Accordingly, accurate tumor staging is indispensable to initiate optimal therapy regime to achieve best oncological outcome (2). A reliable risk stratification is crucial to triage patients and predict survival. Contrast-enhanced computed tomography (CT) scan of the chest, abdomen and pelvis including urography is the mainstay of imaging used for tumor staging (6). According to the guidelines of the European Association of Urology (EAU) LN larger than 8 mm in the shorter-axis in the pelvis should be considered as pathologic (7). Despite the guidelines detection of positive LN is still insufficient with a sensitivity of only 48-87% (7). Magnet resonance imaging (MRI), dynamic imaging such as 18F-FDG-PET/CT and 11C-Choline PET have shown similar results in LN staging compared to conventional CT scan (8).

Accordingly, patients with suspicion for LN metastasis in CT scan present a patients group with uncertain risk of progression. Therefore, we re-evaluated CT scan characteristics of patients with clinical lymph node-positive status in detail to predict LNR and oncological outcome. Furthermore, the invention of a CT scan based risk stratification might improve clinical therapy management of cN+ patients.

## MATERIAL AND METHODS

Between 2004 and 2019, a total of 1565 patients underwent RC, 1127 due to UCB. Follow-up was completed in 764 (67.8%) patients without suspect lymph nodes (cN0). 249 patients presented with clinical suspicious lymph nodes (cN+); of those in 166 (66.7%) patients complete preoperative CT imaging and follow-up was available. At our institute indication for RC were MIBC, BCG-refractory NMIBC after exclusion of distant disease (cM0) or palliative reasons, according to the guidelines of the EAU. RC was performed with a standardized surgical procedure and included pelvic LAE. Urinary diversion was either assured by ileal neobladder, ileal conduit or ureterocutaneostomy. Histological specimen was worked up by our experts for urogenital malignancies. Classification was performed on the report of latest TNM Classification of Malignant Tumors and UICC-classification. Post-operative rehabilitation was offered to all patients.

All patients underwent multi-institutional contrast-enhanced abdominal/pelvic CT scan with a maximum of 6 weeks prior surgery. The slice thickness used to reconstruct images for retrospective review was 5.0mm.

**Figure 1:**
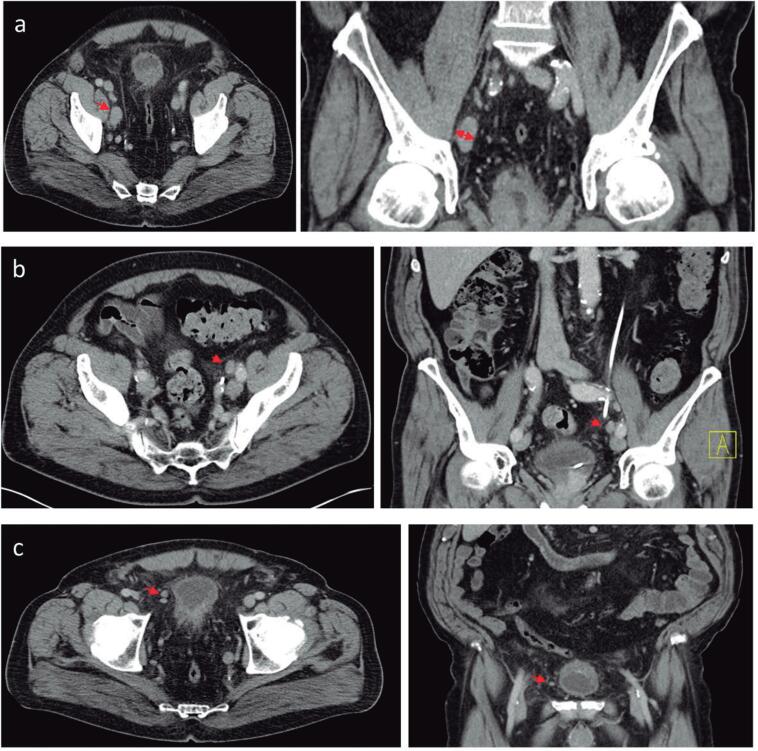
Morphological characteristics of lymph nodes in CT scan Representative pelvic CT scan showing lymph node characteristics such as size (A), morphology (B) and loco-regional number (C) in transversal and coronal plane.

**Figure 2:**
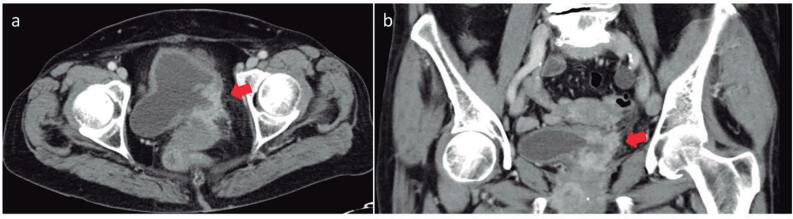
Morphological characteristic of locally advanced tumor stage in CT scan Representative pelvic CT scan showing locally advanced tumor stage in transversal plane (A) and coronal plane (B).

Image analysis was conducted by one radiologist specialized in genitourinary imaging. Images were reviewed on Picture Archiving and Communication System (PACS). To analyse the metastatic involvement of LNs following parameters on CT were observed: size >15mm in the short axis. Morphology of LN (MLN), the LN were considered as suspicious with the loss of the fatty hilum and the normal reniform LN shape with a more rounded or irregular configuration. Also, number of loco-regional LN (NLN) were evaluated (Figure-1). Increased numbers were seen as metastatic. Additionally, local tumor stage was differentiated in organ-confined and locally advanced (Figure-2). According to these characteristics in preoperative CT scan we developed a scoring system to predict oncological outcome.

CT scan characteristics were investigated concerning their impact of lymph node ratio (LNR), cancer specific survival (CSS) and overall survival (OS). Cut-off for significant LNR was >0.2 according to the literature (5).

The scoring system is based on the criteria in CT scan mentioned above (LN size >15mm, increased NLN, suspicious MLN, advanced local tumor stage). Each criteria holds equal value when positive. Patients with cN+ fulfilling ≤2 positive criteria were defined as low-risk group and patient fulfilling ≥3 positive criteria as high-risk group. The control group were cN0 patients either with organ-defined tumor stage and locally advanced tumor stage in CT scan.

Follow-up was performed according to the guidelines of the EAU and carried out either by our outpatient clinic or office-based urologists. Moreover, specific questionnaires regarding quality of life (QoL), complications due to urinary diversion and oncologic follow-up including reports of follow-up imaging were sent twice in the first year after RC and then once a year. Median follow-up time for all UCB patients was 24 months and for our cohort (clinical lymph node-positive patients) 14 months.

Written informed consent was obtained from all patients following the World Medical Association Declaration of Helsinki (9). The institutional review board (Ethikkomission der Ludwig-Maximilian-Universität München) approved the study design prior initiation of the study (Reference number 20-179).

Statistical analysis on outcome was performed by the Kaplan-Meier method and log-rank test. Continuous data were compared using the Mann-Whitney U test. P values below 0.05 were regarded as significant. All calculations were performed using MedCalc 19 software (MedCalc, Ostend, Belgium).

## RESULTS

This study included an investigation cohort of 166 patients with suspicious LN (cN+) and a control group of 764 patients with no signs of LN metastasis (cN0) in preoperative CT scan. There were no significant differences in sex and age (Table-1).

**Table 1:** Patient characteristics.

		cN0 patients		cN+ patients		p-value
		(n=764)		(n=166)		
**Age [years]**						0.306
	Median	70		69		
	IQR	63 – 77		70 – 77		
		n	%	n	%	
**Gender**						0.562
	Male	587	77	131	79	
	Female	177	23	35	21	
**pT**						<0.001
	pTX	12	2	3	2	
	pT0	73	10	5	3	
	pTa/is	131	17	20	12	
	pT1	66	9	10	6	
	pT2	170	22	30	18	
	pT3	243	32	60	36	
	pT4	69	9	38	23	
**pN**						<0.001
	pN0	531	79	83	54	
	pN+	138	21	71	46	
**M**						<0.001
	M0	725	95	135	81	
	M1	39	5	31	19	

The cN+ cohort showed positive CT scan characteristics as following: 121 (72.9%) suspicious morphology; 44 (26.5%) increased number of LNs; 36 (21.7%) LN size >15mm. Histopathological LN metastasis were confirmed in 46% of cN+ cohort. For cN0 patients final histopathological specimen revealed in 21% LN metastasis.

Distribution of local tumor stage in preoperative CT scan was reported in cN+ cohort with 40.4% organ-confined and 55.4% with locally advanced disease. cN0 cohort showed 24% with locally advanced tumor stage.

Accordingly, overall accuracy for local tumor stage was 62% and for detection of LN metastasis 72%.

### Lymph node ratio

Mean of significant LNR (>0.2) for cN0 and cN+ patients were 4.6% and 19.5%. Mean of total removed LN was 14.51 in cN0 and 14.58 in cN+. Mean of pathological confirmed LN metastasis was 0.61 in cN0 and 2.26 in cN+. Overall, we described significantly higher LNRs in cN+ than in cN0 patients (p <0.001). LN size >15mm was associated with significantly increased LNR (p=0.002). In contrast, MLN (p=0.360) and NLN (p=0.440) showed no impact as single positive criteria on LNR. Furthermore, local tumor staging in preoperative CT scan either in cN0 and cN+ patients showed no correlation to higher LNRs (p=0.296; p=0.432). These results are visualized in Table-2.

**Table 2:** Impact of CT scan characteristics on LNR.

		Mean	95-Confidence Intervall	p-value
**LN size**				
	>15mm	0.277	0.168 – 0.385	0.002
	<15mm	0.147	0.098 – 0.196	
**LN morphology**				
	Suspicious	0.172	0.122 – 0.221	0.360
	Normal	0.199	0.084 – 0.315	
**LN loco-regional**				
	Increased	0.257	0.154 – 0.361	0.440
	Normal	0.148	0.100 – 0.196	
**Local tumor stage**				
	Advanced	0.215	0.157 – 0.273	0.432
	Organ-confined	0.184	0.127 – 0.240	

Single CT scan characteristics have been analyzed to hold potential to predict significantly increased LNR. LNR cut off was defined >0.2. Only LN larger 15mm in diameter have shown to be associated with increased LNR (p=0.002).

### Oncological outcome

Five-year CSS of investigation cohort cN+ were 60% for clinical organ-confined disease and 49% for clinical local advanced disease. For control cohort of cN0 patients we reported a 5-year CSS of 62% for clinical organ-confined disease and 50% for clinical local advanced disease.

There was no significant correlation of CT scan characteristics and CSS/OS such as LN size >15mm (p=0.50, p=0.90), suspicious MLN (p=0.97, p=0.65) and locally advanced tumor stage (p=0.89; p=0.53) in the cohort of cN+ patients. CSS and OS were significantly reduced for subgroup of increased NLNs in comparison to normal NLNs with a 5-years CSS 32% vs. 52% and 5-years OS 26% vs. 42% (p=0.001; p=0.002).

### Computed-tomography based scoring system

Patients were defined as low-risk group fulfilling up to two positive criteria in CT scan and as high risk group with three and more positive criteria. Five-year CSS and OS in low-risk profile were 48% and 37%. In high-risk profile 5-year CSS and OSS were 34% and 30%.

**Figure 3:**
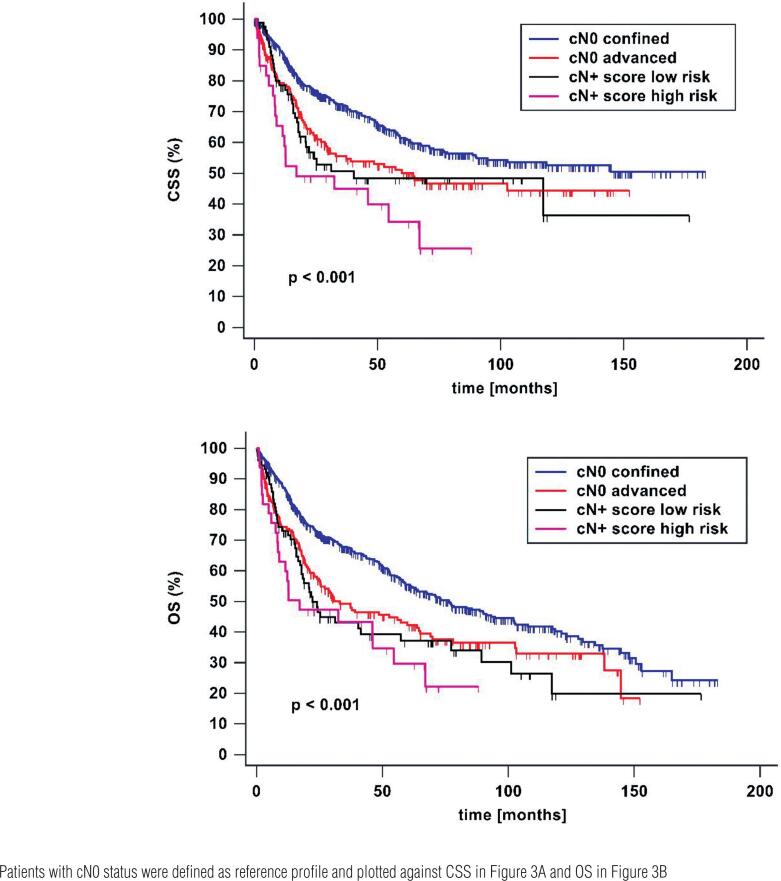
Cancer specific (A) and overall survival (B) after radical cystectomy with regard to lymph node status and local tumor stage in preoperative CT scan CT morphological characteristics such as increased number of lymph nodes, suspicious morphology, size >15mm and locally advanced tumor stage were each defined as one point. All positive criteria were summed up. Patients with ≤2 points were defined as low-risk and patients with ≥3 were stratified as high-risk profile.

As seen in Figure-3 oncological outcome differed highly significant between cN0 organ-confined, cN0 locally advanced, low-risk and high-risk group (p <0.001).

## DISCUSSION

Clinical disease staging in patients with high risk UCB is essential for therapy management (10). CT scan is the mainstay of imaging because of its wide availability, fast feasibility and manageable costs (11). Nevertheless, its accuracy to detect local tumor invasion and LN involvement is still very limited (11). Advanced T-stage and presence of LN metastasis significantly reduce survival of patients (12, 13).

Up to date, evidence of CT morphological criteria is limited to improve detection of LN metastasis in preoperative CT scan. Furthermore, there is no imaging guided risk stratification to adapt treatment management for cN+ patient.

The presented accuracy of detecting local advanced disease and LN metastasis with 62% and 72% is in accordance with the literature (14–17). Therefore, the presented results hold high potential to be widely transferable.

According to our study specific CT morphological characteristics are associated with increased LNR and impaired survival. Conventional TNM classification of LN status has been controversially discussed in regard of predicting oncological outcome (5). Therefore, Herr et al. has reported a simple evaluation of efficacy of LAE and precise prediction of oncological outcome by using the ratio of metastatic LNs to all resected LNs (5). According to our results LNs measured >15mm in CT scan are significantly associated with higher LNR.

Interestingly, EAU guideline already defines LN in the pelvis larger than 8mm as suspicious for nodal metastasis (2). In contrast, normal sized LNs may show histologically metastasis and reactively enlarged LNs reveal no malignant potential (17). It is reported that LN metastasis of bladder cancer leads to limited enlargement (17). Still, LN size remains one of the major criteria to distinguish between normal and suspicious LN (18). According to our findings and the literature, lymph node size should be a criteria to evaluate LNs in CT scan even though size itself presents no reliable criteria (6).

Secondly, LN morphology and number of loco-regional LN were evaluated but revealed no significant impact of LNR. To date there is no evidence that these single characteristics have been independently analyzed before.

Furthermore, we firstly evaluated the number of loco-regional lymph nodes (NLN) as a independent criteria in CT scan on oncological outcome. According to our findings the presence of an increased number of LNs correlates with poorer CSS and OS. This characteristic is seen in other malignancies such as lymphoma which often present groups of LNs in cross-sectional imaging (18, 19). Nevertheless, appearance of groups of LNs is warily to interpret because there are multiple etiologies like inflammatory, infection and malignancies (20). Correspondingly, further research is necessary to confirm these findings in bladder cancer and to analyze pathophysiological mechanism of conglomeration of loco-regional LNs in imaging.

It is well described that CT imaging is very limited in evaluating local tumor expansion in bladder cancer (11). However, locally advanced disease in CT scan is an indicator for poor oncological outcome in cN0 patients according to our study. These findings are in line with the results by Schmid et al. about worse oncological outcome in patients with increased bladder wall thickness (21). It has to be noted by distinguishing the cN0 and cN+ cohorts our data support no prognostic value in the presence of suspicious LNs.

Patients with suspicion of LN metastasis still present a challenging patient group in clinical practice. When LN metastasis are confirmed in histopathological specimen prognosis is poor in contrast to patients who reveal reactive enlarged benign LNs especially with limited T-stage (3). Even rare cases of patients with NMIBC revealing simultaneous distant metastasis have been reported which reflects the large knowledge gap of metastasizing mechanism in UCB (22). This emphasizes the need of preoperative risk stratification for the heterogeneous patients clientele of cN+ patients to adapt treatment option. The presented CT morphological characteristics hold high potential to be used in a practical scoring system to risk stratify cN+. It precisely discriminates a high-risk from a low-risk group and enables comparison to cN0 patients. For various other malignancies scoring systems based on criteria in preoperative CT scans have been developed and been an useful clinical tool (23–25). This useful prediction tool might help to improve best patient management by adapting treatment option for cN+ patients with UCB.

Along this risk stratification therapy management might be adjusted in regard of surgical technique, extend of LAE and use of perioperative chemotherapy therapy. In times of increasing use of robotic surgery evidence rises that independent of T-stage and LN status robotic surgery even with intracorporal urinary diversion can be offered without impairing oncological outcome (26). Nevertheless, imaging guided therapy management might help surgeons to adapt extend of nerve or seminal-sparing resection to improve post-operative QoL (27). In individual situation consideration of seminal- or prostate-sparing surgery can improve post-operative sexual function and QoL (27). Also, high morbidity might encourage to perform bladder preserving therapy (28). Meticulous selection of these potential patients is crucial where precise clinical staging is a key element. Correspondingly, it has been reported that patients undergoing bladder preserving surgery with suspicious LN in preoperative imaging showed poor oncological outcomes (28). This unspecified description of suspicious LN might be complemented by the presented imagine criteria for better triage in this highly specific situation to perform less radical treatment options.

Also adaption of perioperative chemotherapy might be discussable. According to current guideline neoadjuvant chemotherapy should be offered to patients after exclusion of nodal or distance metastasis (7). When pN1 status in RC specimen is confirmed patients have shown better prognosis when treated with adjuvant chemotherapy in comparison to patients who already underwent neo-adjuvant chemotherapy (29). Using a preoperative risk stratification might improve triage of patients with high-risk of pN+ straight to primary surgery and adjuvant treatment for best survival. Further research is needed evaluating imaging guided therapy management on oncological outcome for this demanding patients clientele.

In summary, despite limited accuracy of preoperative CT scan to detect LN metastasis and local tumor stage our findings newly identified impact of single morphological criteria in cN+ patients. To be highlighted is the presence of LN size >15mm in CT is significantly associated with higher LNR and increased number of loco-regional LNs is a prognostic parameter for poor survival.

A practical CT based scoring system discriminates precisely cN+ patients in a high-risk and low-risk group and might build the basis for imaging guided perioperative management. Further research is needed to confirm CT based scoring system in a multi-institutional and prospective setting. Also imaging guided management to provide best individual therapy has to be further evaluated.

This study has its limitation of being a single-center study and its retrospective character. Also, number of patients and its follow-up period are limited.

## CONCLUSION

Although CT scan is limited in staging of patients with high-risk UCB it still remains as standard of care. Due to its poor accuracy to detect LN metastasis cN+ patients presents a clientele with uncertain risk of progression. Therefore, single CT morphological characteristics have been analyzed and its prognostic value on LNR and survival have been identified.

Along the investigated criteria a CT based scoring system was developed to precisely differentiate high- from low-risk cN+ patients in regard of oncological outcome. This might open the opportunity for individual adapted therapy management to improve best patient care.

## ABBREVIATION

CSS: cancer specific survival
CT: computed tomography
EAU: European Association of Urology
LAE: Lymphadenectomy
LNR: Lymph node ratio
MLN: Morphology of lymph nodes
MIBC: Muscle-invasive bladder cancer
MRI: Magnet-resonance imaging
NLN: Number of lymph nodes
NMIBC: Non-muscle-invasive bladder cancer
OS: Overall survival
QoL: Quality of Life
RC: Radical cystectomy
UCB: Urothelial carcinoma of the bladder
